# Model Embraced Electromechanical Coupling Time for Estimation of Heart Failure in Patients With Hypertrophic Cardiomyopathy

**DOI:** 10.3389/fcvm.2022.895035

**Published:** 2022-06-16

**Authors:** Su Hu, Lan Mi, Jianli Fu, Wangxia Ma, Jingsong Ni, Zhenxia Zhang, Botao Li, Gongchang Guan, Junkui Wang, Na Zhao

**Affiliations:** ^1^Department of Cardiovascular Medicine, Shaanxi Provincial People’s Hospital, Xi’an, China; ^2^Department of Cardiovascular Medicine, The Second Affiliated Hospital of Xi’an Medical University, Xi’an, China; ^3^Key Laboratory of Carcinogenesis and Translational Research (Ministry of Education), Department of Lymphoma, Peking University Cancer Hospital and Institute, Beijing, China; ^4^Department of Cardiovascular Surgery, Shaanxi Provincial People’s Hospital, Xi’an, China; ^5^Department of Cardiovascular Medicine, Zhouzhi County Hospital, Xi’an, China; ^6^Department of Cardiovascular Medicine, Huazhou District People’s Hospital, Weinan, China; ^7^Department of Cardiovascular Medicine, Pucheng County Hospital, Weinan, China

**Keywords:** hypertrophic cardiomyopathy, heart failure, electromechanical coupling time, scoring app, predictive model

## Abstract

**Objective:**

This study aimed to establish a model embraced electromechanical coupling time (EMC-T) and assess the value of the model for the prediction of heart failure (HF) in patients with hypertrophic cardiomyopathy (HCM).

**Materials and Methods:**

Data on 82 patients with HCM at Shaanxi Provincial People’s Hospital between February 2019 and November 2021 were collected and then formed the training dataset (*n* = 82). Data were used to screen predictors of HF using univariate and multivariate analyses. Predictors were implemented to discover the optimal cut-off value, were incorporated into a model, and shown as a nomogram. The cumulative HF curve was calculated using the Kaplan–Meier method. Additionally, patients with HCM at other hospitals collected from March 2019 to March 2021 formed the validation dataset. The model’s performance was confirmed both in training and validation sets.

**Results:**

During a median of 22.91 months, 19 (13.38%) patients experienced HF. Cox analysis showed that EMC-T courses in the lateral wall, myoglobin, PR interval, and left atrial volume index were independent predictors of HF in patients with HCM. Five factors were incorporated into the model and shown as a nomogram. Stratification of patients into two risk subgroups by applying risk score (<230.65, ≥230.65) allowed significant distinction between Kaplan–Meier curves for cumulative incidence of HF events. In training dataset, the model had an AUC of 0.948 (95% CI: 0.885–1.000, *p* < 0.001) and achieved a good C-index of 0.918 (95% CI: 0.867–0.969). In validation dataset, the model had an AUC of 0.991 (95% CI: 0.848–1.000, *p* < 0.001) and achieved a strong C-index of 0.941 (95% CI: 0.923–1.000). Calibration plots showed high agreement between predicted and observed outcomes in both two datasets.

**Conclusion:**

We established and validated a novel model incorporating electromechanical coupling time courses for predicting HF in patients with HCM.

## Introduction

Hypertrophic cardiomyopathy (HCM) is the most common genetic cardiovascular disorder, which is recognised as an important cause of sudden cardiac death (SCD) and can lead to disability from heart failure (HF) and stroke (1–3). Owing to the improvement of SCD risk stratification and use of implantable defibrillators, HF has become an increasingly prominent adverse outcome in the natural course of HCM (4, 5). Mild-to-severe cardiac functional impairment, commonly expressed as exertional dyspnoea and fatigue, occurs in approximately 50% of patients with HCM. As the left ventricular ejection fraction (LVEF) generally remains within the normal range (6), identification of predictors of HF development in patients with HCM is needed in clinical practice. Few studies have recognised that diastolic function parameters, including mitral inflow, pulmonary venous flow, and global longitudinal strain evaluation by speckle-tracking echocardiography, are associated with HF outcome (7–10). However, data on risk assessment models to monitor the evolution of HF in patients with HCM are lacking.

Cardiac excitation-contraction coupling is a process that links cardiomyocyte action potential to cardiomyocyte contractile (11). The process is difficult to test experimentally; nevertheless, cardiac electromechanical coupling efficiency, presented as electromechanical coupling time, previously evaluated by combining tissue Doppler imaging (TDI) echo and electrocardiogram (ECG) measurement has been proven (12). In a preliminary study, electromechanical coupling time was proposed as a potential index for evaluating cardiac systolic function in patients with stage B HF, which is a precursor of HF (12, 13). Few studies have explored electromechanical coupling time and outcomes in HCM, and we hypothesised that electromechanical coupling time was delayed in HCM with HF and would predict HF in patients with HCM. The current study was undertaken to establish a model based on electromechanical coupling time and to assess the value of the model for the prediction of HF occurring in a cohort of patients with HCM.

## Materials and Methods

### Study Protocol and Patient Involvement

This was an observational, multi-centre cohort study performed at Shaanxi Provincial People’s Hospital, China. The HCM cohort was evaluated to identify consecutive patients from February 2019 to November 2020 and formed the training dataset. An independent population study with an external HCM cohort was performed in Shaanxi, China at Pucheng County Hospital, Zhouzhi County Hospital, and Huazhou District People’s Hospital from March 2019 to March 2021.

Inclusion criteria for the study were as follows: (1) a diagnosis of HCM based on the presence of left ventricular hypertrophy on echocardiography (15 mm), which is not solely explained by abnormal loading conditions, according to the guidelines of the European Society of Cardiology (3); (2) age ≥18 and ≤70 years; (3) left ventricular ejection fraction ≥ 50%; and (4) New York Heart Association (NYHA) function class I or II.

The exclusion criteria were as follows: (1) other cardiac or systemic diseases that may produce left ventricular hypertrophy; (2) a history of hypertension; (3) a history of diabetic mellitus; (4) a history of coronary artery disease; (5) burden of premature beat ≥ 10%, second- or third-degree atrioventricular block, left bundle branch block (LBBB), interventricular conduction delay (IVCD), or acute pulmonary oedema; (6) previous episode of atrial fibrillation; (7) previous septal alcohol ablation or morrow surgery; (8) onset of atrial fibrillation during follow-up; and (9) onset of SCD during follow-up.

Our research focused on the feedback and input of patients through consultations. Patients were involved in the study design and conduction of this research. During the preliminary feasibility stage, the priority of the research question, and methods of recruitment were informed *via* panel discussions with patients and one structured on-line interviews. Furthermore, a patient joined the independent steering committee during the execution phases of the research. We intended to share the vital results of this study with our patients and planned to explore patient and public involvement in the development of an appropriate approach of dissemination.

### Data Collection

Demographic characteristics including age, sex, body mass index (BMI), family history of sudden cardiac death (SCD), unexplained syncope ≤ 2 years, risk-SCD score (3), heart rate (HR), resting blood pressure, 6-min walk test (6-MWT), brain natriuretic peptide (BNP), and medication history were collected at enrolment.

Peripheral blood was sampled from patients in a fasting state in the morning following the enrolment day. Venous plasma concentrations of serum kalium, calcium, natrium, glycated haemoglobin, total cholesterol, low-density lipoprotein, high-density lipoprotein, triglyceride, lipoproteins, serum creatinine, N-terminal pro-B-type natriuretic peptide, red blood cells, haemoglobin, white blood cells, creatine, phosphokinase-myohaemoglobin (MB), myohaemoglobin (MB), troponin I, thyroid stimulating hormone, free triiodothyronine 3, and free triiodothyronine 4 were determined in the clinical laboratory department using standard biochemical techniques.

An ECG was performed as previously described (14). Twenty-four-hour ECG recordings were obtained using a digital Holter system. The transthoracic echocardiography protocol was based on standardised acquisition from the European Association of Echocardiography/American Society of Echocardiography (ASE) guidelines in all patients (15). Recordings in standardised views, including two-dimensional, M-mode, and colour flow Doppler and pulsed tissue Doppler imaging (TDI), were acquired with an EPIQ 7C system (S5-1 probe, Philips, Netherlands) for subsequent analysis. All measurements were calculated as the average of three consecutive cardiac cycles. Interventricular septum (IVS) thickness and left ventricular posterior wall (LVPW) thickness were defined as the greatest thickness in any single segment. Right ventricular diameters (RVDs) were evaluated as the greatest diameter at the end-diastolic stage in the apical four-chamber view and left ventricular long axis view, respectively. Left ventricular mass (LVM) and LVM index (LVMI) were calculated according to the Devereux formula: LVM (g) = 0.8 × 1.04 × [(IVS + LVPW + LVEDD)^3^-LVEDD^3^] + 0.6, LVMI (g/m^2^) = LVM/body surface area (BSA), respectively, and the body surface area (BSA) was calculated as follows: 0.0061 × height (cm) + 0.0128 × weight (kg)–0.1529. Left atrial volume (LAV) was calculated using the biplane area length method at end systole (16), LAVI (ml/m^2^) = LAV/BSA. Left ventricular ejection fraction (LVEF) was calculated using biplane Simpson’s rule (15). Left outflow tract gradients (LVOTG) were measured and automatically calculated from the flow velocities using the modified Bernoulli equation (17). The tricuspid regurgitation peak velocity was recorded using continuous wave Doppler.

**FIGURE 1:**
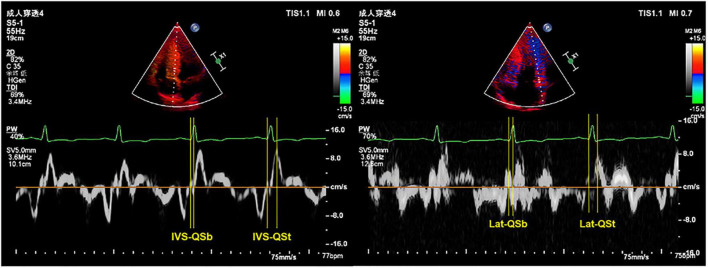
Representative images of TDI combined with ECG to measure the electromechanical coupling time. TDI, tissue Doppler imaging; ECG, electrocardiography; IVS, interventricular septum; Lat, lateral wall; QSb, from the onset of QRS wave on ECG to the beginning of S wave on TDI; QSt, from the onset of QRS wave on ECG to the top of S wave on TDI.

Electromechanical coupling time courses (Qsb, Qst) in the interventricular septum (IVS) and lateral wall (Lat) of the left ventricle were measured using a combination of TDI echo and ECG. The Qsb time course is from the onset of the Q wave on the ECG to the beginning of the S wave on the TDI. The Qst time course is from the onset of the Q wave on the ECG to the top of the S wave on the TDI (Figure 1). HR is related to the electrical and mechanical activities of the heart (18). We determined all the data of electromechanical coupling time courses with HR correction, referenced as the formula in a previous study: electromechanical coupling time courses with HR correction = electromechanical coupling time courses without HR correction/HR × 60 (12, 18).

### End-Point

The primary end-point was HF admission, which was defined as follows: (1) exertional dyspnoea, (2) NYHA function class III-IV, and (3) diuretic is essential for improving symptoms. All patients were followed-up *via* face-to-face interviews in our hospital. The duration of follow-up was determined using the initial visit to the date of the first HF occurrence or the last visit (December 2021).

### Statistical Analysis

Data were collected using IBM SPSS statistical software (version 20.0) for Windows (SPSS Inc., Chicago, IL, United States) and R 4.0.1 (New York^1^). Continuous variables were expressed as the mean and standard deviation. Categorical variables were expressed as frequencies and percentages. The Kolmogorov–Smirnov test was used to assess the normal distribution of the quantitative variables. The independent samples *t*-test was performed to compare parametric values between the HF and non-HF groups, whereas categorical variables were compared using the Chi-square test. Univariate and multivariate survival analyses were performed using the Cox regression analysis. Interclass correlation coefficient (ICC) was used to evaluate intra- and inter-observer variability in electromechanical coupling time parameters. The clinically significant predictors of the final regression model were formulated as a nomogram. Kaplan–Meier survival curves were used in the two groups according to the model score to further assess the prognostic value. The function “surv_cutpoint” in the R package “survminer” was applied to determine the optimal cut-off value of these chosen variables in our study. The accuracy of the predictive model was assessed using receiver operating characteristic curve (ROC) analysis and Harrell’s concordance index (C index). A calibration curve was derived to explore the consistency between predicted probabilities by the nomogram and observed HF rates. External validation was performed in an independent population. The nomogram was used to assess each patient in the validation dataset. Thereafter, the area under the curve (AUC), C-index and calibration curve were calculated to assess the discriminative performance and predictive accuracy of the nomogram model. Decision curve analysis (DCA) was used to calculate the net benefit by using the model in both training and validation datasets (19). The total scores of each patient were calculated based on the nomogram, and we developed an application for clinical use of the model.

All probability values were two-tailed. A *p*-value of less than 0.05 was considered statistically significant. The R code is submitted as a Supplementary Material.

### Ethics

Our study complied with the Declaration of Helsinki and was approved by the ethics committee of the Shaanxi Provincial People’s Hospital. Written informed consent was obtained from all patients.

## Results

### Study Population

**FIGURE 2:**
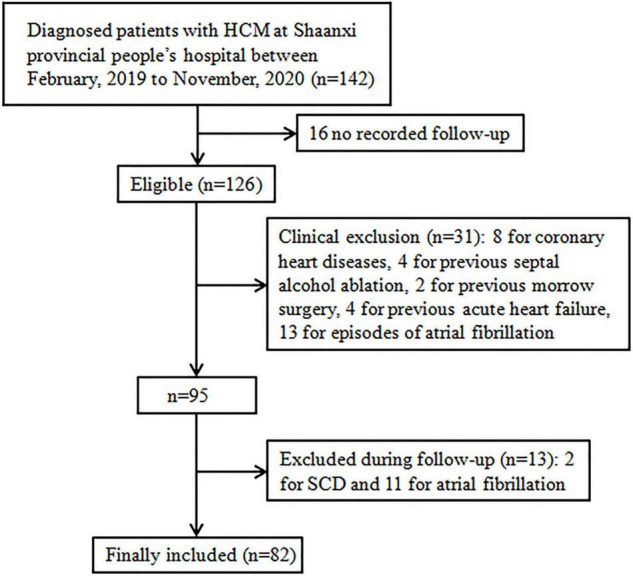
Flow chart. Study selection process: over 142 patients were diagnosed with HCM at Shaanxi Provincial People’s Hospital between February, 2019 and November, 2020 (*n* = 142), 16 did not have available follow-up data, 8 for coronary heart diseases, 4 for previous septal alcohol ablation, 2 for previous morrow surgery, 4 for previous acute heart failure, 2 for SCD, 10 for previous episode of atrial fibrillation, 11 for onset of atrial fibrillation during follow-up, and 2 for onset of SCD during follow-up; HCM, hypertrophic cardiomyopathy, SCD, sudden cardiac death.

Eighty-two patients fulfilled the study inclusion criteria out of a cohort of 142 patients with HCM. The reasons for exclusion were: 16 for having no second visit during the study period (lost to follow-up), 8 for coronary heart diseases, 4 for previous septal alcohol ablation, 2 for previous morrow surgery, 4 for previous acute HF, 2 for SCD, 10 for previous episode of atrial fibrillation, 11 for onset of atrial fibrillation during follow-up, and 2 for onset of SCD during follow-up (Figure 2). Clinical, demographic, echocardiographic characteristics, and electromechanical coupling time parameters of the population at the initial evaluation are presented in Table 1. The mean age was 42.20 ± 10.83 years, and 46 (56.09%) patients were men. The left ventricular wall thickness was 22.60 ± 5.04 mm (range, 15–35), and the mean ejection fraction was 59.50 ± 5.72%. All patients received standardised medical care during the clinical course.

**TABLE 1:** Baseline characteristics of patients with and without heart failure (*n* = 82).

Variable	All patients (*n* = 82)	HF (*n* = 19)	NHF (*n* = 63)	*P*
Age (years)	42.2010.83	**38.47 ± 10.17**	**43.32 ± 10.85**	0.008**
Male [*n* (%)]	46 (56.10%)	11 (57.90%)	35 (55.60%)	0.858
BMI (Kg/m^2^)	22.76 ± 1.77	22.72 ± 1.54	22.78 ± 1.84	0.912
Smoking [*n* (%)]	29 (35.37%)	10 (52.60%)	19 (30.16%)	0.074
Drinking [*n* (%)]	23 (28.05%)	**9 (47.37%)**	**14 (22.22%)**	0.034*
Hypertension [*n* (%)]	17 (20.73%)	6 (31.58%)	11 (17.46%)	0.186
Diabetes [*n* (%)]	11 (13.41%)	2 (10.53%)	9 (14.29%)	0.675
CA [*n* (%)]	10 (12.20%)	3 (15.79%)	7 (11.11%)	0.587
SCD family history	15 (18.29%)	6 (31.50%)	9 (14.20%)	0.171
History of syncope	36 (43.90%)	9 (47.30%)	27 (42.90%)	0.933
SCD score	3.27 ± 1.63	2.77 ± 1.06	3.42 ± 1.75	0.131
6MWT (m)	355.85 ± 91.80	**394.74 ± 81.47**	**344.13 ± 92.08**	0.034*
HR1 (beats/min)	75.24 ± 9.29	73.47 ± 8.80	75.78 ± 9.44	0.347
HR2 (beats/min)	73.59 ± 7.89	77.00 ± 12.63	72.89 ± 6.98	0.071
SBP (mmHg)	121.83 ± 7.75	122.16 ± 6.39	121.73 ± 8.16	0.834
DBP (mmHg)	75.76 ± 5.77	75.16 ± 5.68	75.94 ± 5.82	0.609
β*-blocker (metoprolol)*				
*Non*β*-blocker*	*23 (28.05%)*	*7 (36.84%)*	*16 (25.40%)*	*0.333*
*47.5 mg qd*	*11 (13.41%)*	*4 (21.05%)*	*7 (11.11%)*	*0.265*
*90.0 mg qd*	*12 (14.64%)*	*3 (15.79%)*	*9 (14.29%)*	*0.871*
≤*6 (months) [n (%)]*	*9 (10.98%)*	***0 (0.00%)***	***9 (14.29%)***	0.002**
*47.5 mg qd*	*4 (4.88%)*	*0 (0.00%)*	*4 (6.35%)*	*0.260*
*90.0 mg qd*	*5 (6.10%)*	*0 (0.00%)*	*5 (7.94%)*	*0.585*
*6*∼*24 (months) [n (%)]*	*17 (20.73%)*	*5 (26.31%)*	*12 (19.05%)*	*0.496*
*47.5 mg qd*	*7 (8.54%)*	*2 (10.52%)*	*5 (7.94%)*	*0.723*
*90.0 mg qd*	*10 (12.19%)*	*3 (15.79%)*	*7 (11.11%)*	*0.585*
≥*24 (months) [n (%)]*	*33 (40.24%)*	*7 (36.84%)*	*26 (41.27%)*	*0.732*
*47.5 mg qd*	*12 (14.63%)*	*3 (15.79%)*	*9 (14.29%)*	*0.871*
*90.0 mg qd*	*21 (25.61%)*	*4 (21.05%)*	*17 (26.98%)*	*0.604*
WBC count (10^9^/L)	6.13 ± 1.39	5.89 ± 1.58	6.21 ± 1.33	0.387
RBC count (10^12^/L)	4.52 ± 0.58	4.48 ± 0.36	4.53 ± 0.63	0.750
HB (g/L)	136.61 ± 14.88	137.11 ± 12.72	136.46 ± 15.56	0.870
PLT count (10^12^/L)	198.67 ± 63.20	188.84 ± 60.75	201.63 ± 64.09	0.443
ALT (U/L)	25.89 ± 11.52	29.26 ± 10.69	24.87 ± 11.65	0.146
AST (U/L)	26.90 ± 9.41	28.47 ± 12.51	26.43 ± 8.32	0.410
TBiL (umol/L)	14.24 ± 5.43	13.95 ± 5.10	14.32 ± 5.57	0.797
DBiL (umol/L)	5.08 ± 2.30	5.14 ± 2.43	5.06 ± 2.28	0.893
K^+^ (mmol/L)	4.22 ± 0.49	4.17 ± 0.50	4.23 ± 0.50	0.674
Na^+^ (mmol/L)	140.82 ± 3.07	140.58 ± 4.35	140.89 ± 2.61	0.702
Cl^–^ (mmol/L)	103.57 ± 4.65	102.53 ± 5.33	103.89 ± 4.42	0.265
Ca^2+^ (mmol/L)	2.25 ± 0.10	2.27 ± 0.09	2.24 ± 0.10	0.239
GLU (mmol/L)	6.11 ± 0.93	6.27 ± 1.05	6.05 ± 0.89	0.374
CREA (umol/L)	66.60 ± 14.34	**73.22 ± 10.34**	**64.60 ± 14.84**	0.021*
UA (umol/L)	344.59 ± 73.33	344.00 ± 79.21	344.77 ± 72.13	0.968
UREA (mmol/L)	6.02 ± 2.19	5.83 ± 2.63	5.85 ± 2.03	0.151
TG (mmol/L)	1.40 ± 0.50	**1.63 ± 0.57**	**1.34 ± 0.45**	0.022*
TC (mmol/L)	3.42 ± 1.11	3.18 ± 1.13	3.49 ± 1.11	0.297
HDL (mmol/L)	1.20 ± 0.35	1.20 ± 0.32	1.20 ± 0.36	0.986
LDL (mmol/L)	2.28 ± 0.51	2.22 ± 0.55	2.30 ± 0.50	0.583
ApoA1 (g/L)	1.20 ± 0.15	1.25 ± 0.14	1.19 ± 0.14	0.074
ApoB (g/L)	0.92 ± 0.41	0.91 ± 0.17	0.92 ± 0.46	0.901
CK-MB (ng/ml)	3.74 ± 1.48	3.80 ± 1.86	3.72 ± 1.37	0.841
TnI (ng/ml)	0.15 ± 1.10	0.03 ± 0.03	0.19 ± 1.26	0.580
MB (ng/ml)	56.02 ± 104.72	94.11 ± 213.04	44.58 ± 23.92	0.070
PT (sec)	13.81 ± 2.34	14.22 ± 3.48	13.68 ± 1.89	0.383
PTR	1.07 ± 0.17	1.09 ± 0.26	1.06 ± 0.13	0.448
PTINR	1.14 ± 0.23	1.19 ± 0.35	1.12 ± 0.19	0.273
APTT (sec)	33.06 ± 5.00	34.30 ± 5.96	32.68 ± 4.67	0.220
Fg (g/L)	3.13 ± 0.92	2.99 ± 0.90	3.18 ± 0.93	0.442
TT (sec)	17.18 ± 2.09	17.41 ± 2.41	17.12 ± 2.00	0.601
FDP (mg/L)	2.46 ± 1.63	2.38 ± 1.34	2.48 ± 1.72	0.352
DD (mg/L)	0.29 ± 0.18	0.28 ± 0.19	0.29 ± 0.18	0.858
TSH (uIU/ml)	4.93 ± 11.34	3.70 ± 1.80	5.30 ± 12.90	0.595
FT3 (pmol/L)	5.68 ± 3.48	5.33 ± 3.66	5.78 ± 3.44	0.617
FT4 (pmol/L)	14.49 ± 4.39	14.85 ± 4.32	14.38 ± 4.44	0.682
BNP (pg/ml)	165.38 ± 65.31	166.68 ± 63.84	164.98 ± 66.25	0.922
HbAlc (%)	5.97 ± 0.70	5.99 ± 0.73	5.92 ± 0.59	0.698
RVD1 (mm)	23.79 ± 3.08	**25.21 ± 2.89**	**24.16 ± 2.48**	0.021*
RVD2 (mm)	24.71 ± 2.91	**26.53 ± 3.53**	**24.19 ± 2.50**	0.001**
LAD1 (mm)	39.16 ± 6.25	40.68 ± 5.96	38.70 ± 6.31	0.227
LAD2 (mm)	42.07 ± 6.22	43.42 ± 5.59	41.67 ± 6.39	0.284
SV (ml)	51.61 ± 6.76	52.63 ± 4.99	51.30 ± 7.21	0.455
LVEF (%)	59.50 ± 5.72	56.84 ± 6.99	59.84 ± 6.06	0.072
IVS (mm)	22.60 ± 5.04	22.37 ± 5.65	22.67 ± 4.89	0.823
LVPW (mm)	11.76 ± 1.32	11.42 ± 1.50	11.86 ± 1.26	0.209
LVM (g)	333.35 ± 65.28	339.84 ± 63.30	331.40 ± 66.24	0.624
LVMI (g/m^2^)	184.92 ± 37.24	182.17 ± 39.39	185.75 ± 36.86	0.716
Peak E (cm/s)	84.59 ± 13.59	85.95 ± 11.62	84.17 ± 14.20	0.621
Peak A (cm/s)	43.78 ± 9.47	42.32 ± 7.20	44.22 ± 10.06	0.445
E/A	1.98 ± 0.30	2.06 ± 0.28	1.95 ± 0.30	0.148
IVS-e′ (cm/s)	5.61 ± 0.92	5.30 ± 0.83	5.70 ± 0.94	0.095
Lat-e′ (cm/s)	7.19 ± 1.28	7.04 ± 1.20	7.24 ± 1.31	0.554
E/e′	13.6 ± 2.88	14.10 ± 2.31	13.26 ± 3.02	0.268
TRVmax (m/s)	3.09 ± 0.53	3.09 ± 0.56	3.08 ± 0.53	0.947
LAVI (ml/m^2^)	39.48 ± 5.74	**43.26 ± 6.46**	**38.33 ± 5.02**	0.001**
LOVTG1 (mmHg)	16.74 ± 7.79	18.26 ± 8.38	16.29 ± 7.61	0.335
LOVTG2 (mmHg)	29.65 ± 11.21	30.95 ± 11.67	29.25 ± 11.13	0.567
*LVOT obstruction*				
*Non-obstruction*	*51 (62.20%)*	*11 (57.89%)*	*40 (63.49%)*	*0.898*
*Occult-obstruction*	*20 (24.39%)*	*5 (26.31%)*	*15 (23.80%)*	*0.900*
*Obstruction*	*11 (13.41%)*	*3 (15.80%)*	*8 (12.71%)*	*0.647*
Vmax1 (cm/s)	189.52 ± 81.88	199.00 ± 91.76	186.67 ± 79.24	0.568
Vmax2 (cm/s)	334.56 ± 76.40	328.95 ± 78.15	336.25 ± 76.42	0.717
IVS-QSb (ms)	68.83 ± 7.39	**75.21 ± 4.98**	**66.90 ± 6.92**	<0.001***
IVS-QSt (ms)	92.51 ± 12.17	**103.68 ± 10.66**	**89.14 ± 10.52**	<0.001***
Lat-QSb (ms)	85.44 ± 7.58	**90.89 ± 6.47**	**83.79 ± 7.14**	<0.001***
Lat-QSt (ms)	107.74 ± 11.18	**124.53 ± 3.88**	**102.68 ± 6.82**	<0.001***
P (ms)	98.57 ± 12.26	102.53 ± 6.99	97.38 ± 13.27	0.109
QRS (ms)	107.34 ± 9.24	110.84 ± 8.49	106.29 ± 9.26	0.059
PR (ms)	151.29 ± 16.95	147.37 ± 17.58	152.48 ± 16.72	0.252
QT (ms)	395.82 ± 16.84	397.79 ± 17.71	395.22 ± 16.66	0.563
QTc (ms)	447.79 ± 32.15	448.42 ± 38.37	447.60 ± 30.38	0.923
QRS axis	8.05 ± 45.53	5.89 ± 50.27	8.70 ± 44.42	0.816
RV_5_ + SV_1_ (mv)	3.51 ± 0.53	3.43 ± 0.54	3.54 ± 0.53	0.450
Total HR (beats)	81643.22 ± 6058.92	83522.21 ± 6318.71	81076.54 ± 5912.77	0.124
Average HR (beats/min)	66.65 ± 5.19	64.74 ± 5.57	67.22 ± 4.98	0.067
Minimum.HR (beats/min)	52.41 ± 9.62	52.16 ± 8.88	52.49 ± 9.90	0.895
Maximum.HR (beats/min)	103.16 ± 8.20	101.68 ± 11.25	103.60 ± 7.09	0.375
NSVT (frequency)	16.80 ± 10.31	13.58 ± 6.64	17.73 ± 10.81	0.120

*HF, heart failure; NHF, none-heart failure; BMI, body mass index; CA, coronary atherosclerosis (coronary artery stenosis < 50%); SCD, sudden cardiac death; 6 MWT, 6 min walk test; HR1, heart rate at enrolment; HR2, heart rate during measurement; SBP, systolic blood pressure; DBP, diastolic blood pressure; WBC, white blood cell; RBC, red blood cell; Hb, haemoglobin; PLT, platelet; ALT, alanine aminotransferase; AST, aspartate aminotransferase; TBiL, total bilirubin; DBiL, direct bilirubin; K^+^, Kalium ion; Na^+^, sodium ion; Cl^–^, chlorine ion; Ca^2+^, calcium ion; GLU, glucose; CREA, creatinine; UA, uric acid; TG, triglyceride; TC, total cholesterol; HDL, high-density lipoprotein; LDL, low-density lipoprotein; ApoA1, apolipoproteinA1; ApoB, apolipoprotein B; CK-MB, creatine kinase isoenzyme; TnI, troponin I; MB, myoglobin; PT, prothrombin time; PTR, prothrombin time ratio; PT-INR, prothrombin time-International normalised ratio; APTT, activated partial thromboplastin time; Fg, fibrinogen; TT, thrombin time; FDP, fibrinolytic products; DD, d-dimer; TSH, thyroid stimulating hormone; FT3, free T3; FT4, free T4; BNP, brain natriuretic peptide; HbAlc, glycated haemoglobin; RVD1, anteroposterior diameter of the right ventricle; RVD2, right ventricular transversal diameter; LAD1, anteroposter left atrial diameter; LAD2, left atrial dimension; SV, stroke volume; LVEF, left ventricular ejection fraction; LVPW, left ventricular posterior wall; LVM, left ventricular mass; LVMI, left ventricular mass index; E/A, peak E/peak A; IVS-e′, interventricular septum-e′; Lat-e′, lateral wall of left ventricle e′; E/e′, mitral valve annulus tissue movement, E/e′; TRVmax, tricuspid regurgitation peak velocity max; LAVI, left atrial volume index; LOVTG1, left ventricular outflow tract gradient at rest; LOVTG2, left ventficular outflow tract gradient when performing the Valsalva manoeuvre; Vmax1, maximum velocity of the left ventficular outflow tract at rest; Vmax2, maximum velocity of the left venticular outflow tract when performing the Valsalva manoeuvre; IVS, intervicular septum; Lat, lateral wall; QSb, from the onset of QRS wave on ECG to the beginning of S wave on TDI; QSt, from the onset of the QRS wave on ECG to the top of the S wave on TDI; Total HR, 24-h total heart rate; Average HR, 24-h average heart rate; Minimum HR, minimum heart rate in 24 h; Maximum HR, maximum heart rate in 24 h; NSVT, non-sustained ventricular tachycardia.*

*P < 0.05, **P < 0.01, ***P < 0.001. Bold and italic values represent significant difference between two groups.

### Baseline Characteristics of Patients With and Without Heart Failure

Patients were followed-up for a median duration of 23 months (interquartile range, 11–34 months). Nineteen patients (23.17%) experienced HF. The baseline characteristics of patients with and without HF are outlined in Table 1. There were no significant differences in BMI, HR, IVS thickness, LVEF, LVOTG at rest, and provoked LVOTG. Compared with patients without HF, those who experienced HF had lower age, higher levels of creatine and triglyceride, obviously longer results for the 6-MWT, largest anteroposterior diameter of the right ventricle (RVD-1), larger right ventricular transverse diameter (RVD-2), and greater left atrial volume index (LAVI). Moreover, all four cardiac electromechanical coupling time courses (IVS-QSb, IVS-QSt, Lat-QSb, and Lat-QSt) were longer in patients who experienced HF (*p* < 0.001 for all comparisons).

### Electromechanical Coupling Time Parameters as Independent Predictors of Heart Failure Event

Univariate Cox analyses to predict the endpoint are shown in Table 2. Significant predictors of HF events were IVS-QSb, IVS-QSt, Lat-QSb, Lat-QSt, SCD family history, 6-MWT, CREA, ApoA1, MB, RVD-1, RVD-2, LVEF, LAVI, P, QRS, and PR. Table 3 presents the results of multivariate Cox analysis, which demonstrated that Lat-QSb [hazard ratio (HR): 1.330; 95% confidence interval (95% CI): 1.083–1.633; *p* = 0.007], Lat-QSt (HR: 1.230; 95% CI: 1.026–1.463; *p* = 0.025), MB (HR: 1.004; 95% CI: 1.000–1.008; *p* = 0.042), PR (HR, 0.925; 95% CI, 0.864–0.989; *p* = 0.023), and LAVI (HR: 1.210; 95% CI: 1.006–1.454; *p* = 0.043) were significant and independent predictors of HF occurrence.

**TABLE 2:** Univariate Cox analysis for HF.

	HR	95% CI	*P*
IVS-QSb	1.180	1.090–1.270	0.000***
IVS-QSt	1.070	1.030–1.110	0.000***
Lat-QSb	1.120	1.050–1.190	0.000***
Lat-QSt	1.160	1.100–1.220	0.000***
SCD family history	2.930	1.110–7.720	0.030*
6MWT	1.000	1.000–1.010	0.044*
CREA	1.040	1.010–1.080	0.021*
ApoA1	71.300	2.310–2200.000	0.015*
MB	1.002	1.000–1.004	0.042*
RVD1	1.200	1.040–1.390	0.015*
RVD2	1.300	1.130–1.490	0.000***
LVEF	0.896	0.820–0.978	0.014*
*P* wave	1.060	1.010–1.120	0.029*
QRS	1.110	1.040–1.190	0.002**
PR	0.969	0.945–0.995	0.018**

*HF, heart failure; IVS, interventricular septum; Lat, lateral wall; QSb, from the onset of QRS wave on ECG to the beginning of the S wave on TDI; QSt, from the onset of the QRS wave on ECG to the top of the S wave on TDI; SCD, sudden cardiac death; 6 MWT, 6-min walk test; CREA, creatinine; ApoA1, apolipoproteinA1; MB, myoglobin; RVD1, anteroposterior diameter of the right ventricle; RVD2, right ventricular transversal diameter; LVEF, left ventricular ejection fraction.*

*P < 0.05, **P < 0.01, ***P < 0.001.

**TABLE 3:** Multivariate Cox analysis for HF.

	HR	95% CI	*P*
Lat-QSb	1.330	1.083–1.633	0.007**
Lat-QSt	1.230	1.026–1.463	0.025*
MB	1.004	1.000–1.008	0.048*
LAVI	1.210	1.006–1.454	0.043*
PR	0.925	0.864–0.989	0.023*

*Lat, lateral wall; QSb, from the onset of the QRS wave on ECG to the beginning of S wave on TDI; QSt, from the onset of the QRS wave on ECG to the top of the S wave on TDI; MB, myoglobin; LAVI, left atrial volume index.*

*P < 0.05, **P < 0.01.

#### Intraobserver and Interobserver Variability

Additionally, the assessment of intra- and inter-observer variability in electromechanical coupling time parameters documented good ICCs. ICCs of all these parameters (IVS-QSb, IVS-QSt, Lat-QSb, Lat-QSt) were 0.827 (95% CI: 0.756–0.881; *p* < 0.001).

#### Predictive Nomogram for the Probability of Heart Failure

**FIGURE 3:**
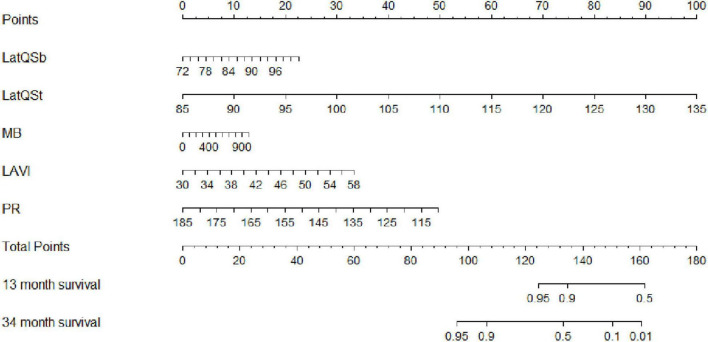
Nomogram to estimate the risk of HF in HCM. To use the nomogram, find the position of each variable on the corresponding axis, draw a line to the points axis for the number of points, add the points from all of the variables, and draw a line from the total points axis to determine the HF probabilities at the lower line of the nomogram. Lat, lateral wall; Qsb, from the onset of QRS wave on ECG to the beginning of S wave on TDI; Qst, from the onset of QRS wave on ECG to the top of S wave on TDI; MB, myoglobin; LAVI, left atrial volume index; PR, PR interval.

Based on the final Cox regression analysis, a nomogram that incorporated independent significant prognostic factors was established (Figure 3). The nomogram illustrated Lat-QSt as sharing the largest contribution to prognosis, followed by PR. Lat-QSb and LAVI had a moderate impact on survival. Each subtype within these variables was assigned a score on a five-point scale. Following the addition of the total score and locating it on the total point scale, a straight line can be drawn down to determine the estimated probability of survival at each time point. In practice, the process has been incorporated into a mobile application (APP)^2^ to conveniently calculate the HF occurrence probability.

**FIGURE 4:**
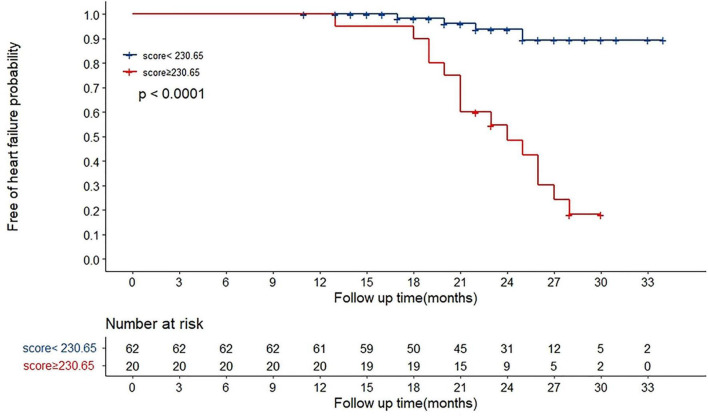
Overall HF occurrence stratified by nomogram in the cohort. The 82 patients were divided into upper and lower groups according to risk scores measured by nomogram (group 1 score: <230.65, group 2 score: ≥230.65). The risk of HF increased along with increasing risk score (log-rank test 28.2, *p* < 0.001).

We determined the cut-off value by grouping the patients into two subgroups after sorting by total score (score: <230.65, ≥230.65). Stratification of patients into two risk subgroups by applying the cut-off value allowed significant distinction between Kaplan–Meier curves for the cumulative incidence of HF events (Figure 4). A log-rank test of the curves for the two patient groups identified significant intergroup differences (log-rank test = 28.2, *p* < 0.001).

### Performance of the Predictive Nomogram

**FIGURE 5:**
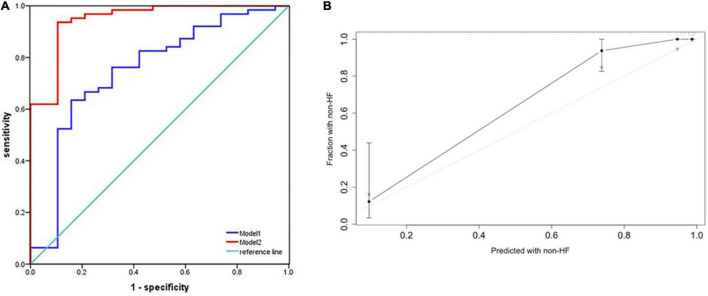
Performance of the predictive nomogram for probabilities of heart failure. **(A)** Receiver operating characteristic (ROC) curve analysis for predicting heart failure. The area under the curve (AUC) for combining MB, PR, and LAVI was 0.751 (95% CI: 0.616–0.886) (Model 1). When Lat-QSb and Lat-QSt were added to the above model (Model 2), the AUC was 0.948 (95% CI: 0.885–1.000, *p* < 0.001). **(B)** Calibration plot of the predictive nomogram for predicting the probabilities of heart failure. An ideal calibration plot is indicated by a 45° diagonal grey line. Lat, lateral wall; QSb, from the onset of Q wave on ECG to the beginning of S wave on TDI; QSt, from the onset of Q wave on ECG to the top of S wave on TDI; MB, myoglobin; LAVI, left atrial volume index; PR, PR interval.

We plotted ROC curves to assess the accuracy of nomogram in predicting the risk of HF occurrence. The AUC for combining MB, PR, and LAVI was 0.751 (95% CI: 0.616–0.886), while that for our predictive nomogram was 0.948 (95% CI: 0.885–1.000, *p* < 0.001) (Figure 5A). Additionally, the nomogram displayed strong discrimination with a C-index of 0.918 (95% CI: 0.867–0.969) and strong calibration. The calibration curve of the accuracy was highly consistent with the diagonal, indicating that the predicted probability of HF was in accordance with the actual probability (Figure 5B).

#### External Validation of the Predictive Nomogram

**FIGURE 6:**
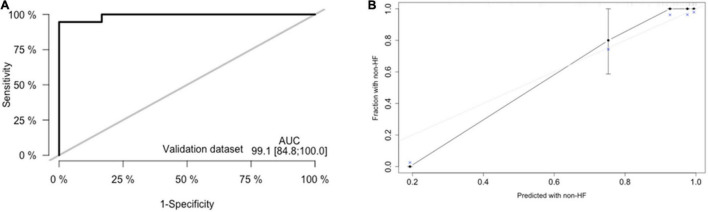
External validation of nomogram in validation dataset. **(A)** Discrimination: Area under the curve (AUC) of the receiver operating characteristic (ROC) curve analysis was 0.948 (95% CI: 0.885–1.000, *p* < 0.001). **(B)** Calibration plot.

External validation was performed through comparisons between the nomogram prediction and actual probability for each patient in an independent population. The clinical, demographic, echocardiographic characteristics as well as electromechanical coupling time parameters of the population at the initial evaluation are presented in Supplementary Table 1. The AUC values and C-index in the validation dataset were 0.991 (95% CI: 0.848–1.000, *p* < 0.001) (Figure 6A) and 0.941 (95% CI: 0.923–1.000), respectively. The calibration plots (Figure 6B) for probabilities of the HF rates presented good concordance between the predicted and observed outcomes.

Both in training dataset and validation dataset, the DCA curves demonstrated the advantages of using predictive nomogram in clinical settings (Supplementary Figure 1).

## Discussion

Since guidelines highlight the evolution of SCD risk stratification strategies to identify patients with SCD and management of left outflow tract (LVOT) obstruction, mortality due to the initiation of SCD and LVOT obstruction has decreased significantly over the years (3, 20). HF development has emerged as a predominant complication in HCM patients; however, there is no recommendation in terms of prevention of HF to meet the needs of patients with HCM. Moreover, few predictive models have been previously explored to predict HF risk in patients with HCM. Our study contributes to the profile of patients with HCM at risk of HF events.

The current cohort study particularly highlights the electromechanical coupling time parameters as independent predictors of HF occurrence in HCM. These time courses represent the efficiency of the electrical activity transferred into mechanical activity, which served as markers of early regional myocardial dysfunction (21). The asymmetry of the heart might influence the electromechanical coupling time; moreover, our study detected electromechanical coupling time course in two walls of the left hypertrophic ventricle (including Lat and IVS). Although all the indexes were significantly longer in HCM patients with HF than in HCM patients without HF, only the electromechanical coupling time courses (Lat-QSb, Lat-QSt) in the lateral wall were associated with the end-point on multivariate analysis. This result might be partly explained by the fact that excitement of the left ventricular lateral wall was usually delayed by 15–20 ms compared with the IVS (22). The association between electromechanical coupling time courses in IVS and HF events attributed to different activation spreads between IVS and the lateral wall, which may be refined by increasing the population sample size.

Additionally, the results revealed that the most significant variables in our model included LAVI, MB, and PR interval. In recent years, LAVI has been recognised as a marker of arrhythmias (23–25), diastolic dysfunction, and haemodynamic load (26) in patients with HCM. Moreover, our study confirmed that LAVI is an independent predictor of HF occurrence in patients with HCM. This result is in line with a few other studies (27), which may shed light on the role of the LA in patients with HCM. The PR interval duration was from the onset of the *P* wave to the beginning of the QRS wave on the ECG. The exclusion criteria for the PR interval in our study were applied as referenced in a previous meta-analysis, which included extreme PR values (≤80 ms or ≥320 ms), second- or third-degree heart block, Wolff–Parkinson–White syndrome, pacemaker placement, use of class I or III blocking medications, and use of digoxin (28). Our results showed that prolonged PR values ranging from 112 to 182 ms (median: 156 ms) were a significant and independent protective factor for HF. This result may be due to the reduced preload influenced by atrial filling time and has not yet been clarified. A previous large-scale meta-analysis identified genetic variants that were significantly associated with PR interval (28). Interestingly, a variant in *MYH6*, which encodes a cardiac myosin heavy chain subunit, leads to human HCM (29, 30). This may provide novel clues to the understanding of atrioventricular conduction for cardiac activity in patients with HCM. The hazard ratio of MB in our model was 1.002, which seems to be slightly affected by HF. This clinical meaning of MB has not been determined, and we also have found that it is difficult to elaborate on the issue.

In the present study, a nomogram incorporating significant predictive factors was established. According to our Kaplan–Meier survival analyses, the estimated cumulative occurrence of HF was higher in the nomogram with a cut-off point ≥ 230.65. Based on the nomogram, Lat-QSt shared the largest contribution to HF occurrence, followed by PR, LAVI, and Lat-QSb. We attempted to compare the models with and without electromechanical coupling time courses. Comparing the model of combining PR, LAVI, and MB and adding up Lat-QSt and Lat-QSb increased the discriminatory predictive value, which was shown by the significant increase in AUC from 0.751 to 0.948 and high Harrell’s C-index (0.918; 95% CI: 0.867–0.969) for predicting HF occurrence in patients with HCM. Moreover, the calibration of the nomogram showed optimal agreement between predictive and actual HF events, guaranteeing the repeatability and reliability of the established model (31). Additionally, external validation further determined the generalisability of our predictive model.

To the best of our knowledge, this is the first model for predicting HF in patients with HCM. Nonetheless, there are several caveats and limitations to be noted. First, HCM patients diagnosed with atrial fibrillation or those with atrial fibrillation within the follow-up period were excluded from the study because of selection bias, and our model may underestimate the occurrence of HF. Second, not all potential predictors, such as the global longitudinal strain mentioned previously (9), provoked LVOT gradient, and genetic phenotypes, were tested for HCM patients in clinical practice. Third, our study was based on data obtained from a single centre, and the model still requires additional databases from other centres to be used for external validation.

## Conclusion

In conclusion, we established and validated a novel model for predicting HF in patients with HCM. Through this model, clinicians could more easily and precisely identify HCM patients at high risk of HF by using the easy-to-use scoring app that we created (see text footnote 2), which might support performance of specific treatment strategies for individual patients.

## Data Availability Statement

The original contributions presented in this study are included in the article/Supplementary Material, further inquiries can be directed to the corresponding author.

## Ethics Statement

Our study complied with the Declaration of Helsinki and was approved by the ethics committee of Shaanxi Provincial People’s Hospital. Written informed consent for participation was not required for this study in accordance with the national legislation and the institutional requirements.

## Author Contributions

SH and JF performed the ultrasound test and collected the data. SH and LM analysed the data and drafted the manuscript. SH and BL prepared and analysed the data. ZZ, JN, WM, GG, and JW provided the original data. NZ conceived and designed the study, interpreted the results, and drafted and revised the manuscript. All authors provided critical comments on the manuscript, read, and approved the final manuscript.

## Conflict of Interest

The authors declare that the research was conducted in the absence of any commercial or financial relationships that could be construed as a potential conflict of interest.

## Publisher’s Note

All claims expressed in this article are solely those of the authors and do not necessarily represent those of their affiliated organizations, or those of the publisher, the editors and the reviewers. Any product that may be evaluated in this article, or claim that may be made by its manufacturer, is not guaranteed or endorsed by the publisher.

## Footnotes

1
http://www.r-project.org/

2
https://lymphomabz.shinyapps.io/DynNomapp/
