# Supplementary material for: Model Embraced Electromechanical Coupling Time for Estimation of Heart Failure in Patients With Hypertrophic Cardiomyopathy
Source: Front Cardiovasc Med. 2022 Jun 16;9:895035. doi: 10.3389/fcvm.2022.895035 (PMC9254680; doi:10.3389/fcvm.2022.895035)
Supplement: Supplementary file 1 [file Table_1.DOCX]

***Supplementary table***

***Supp Table 1*** Baseline characteristics of patients with and without Heart Failure in validation dataset (n=67)

| Variable | All patients(n=67) | HF (n= 13) | NHF (n= 54) | *P* |
| --- | --- | --- | --- | --- |
| Age (years) | 45.70±8.94 | 46.69±8.89 | 45.46±9.02 | 0.660 |
| Male [n (%)] | 34 (30.75%) | 7 (53.85%) | 27(50.00%) | 1.000 |
| BMI (Kg/m^2^) | 24.60±1.86 | 25.40±1.57 | 24.41±1.89 | 0.088 |
| Smoking [n (%)] | 9 (13.43%) | 4 (30.75%) | 5 (9.26%) | 0.112 |
| Drinking [n (%)] | 28 (41.79%) | 8 (61.54%) | 20 (37.04%) | 0.195 |
| Hypertension [n (%)] | 12 (17.91%) | 5 (38.46%) | 7 (12.96%) | 0.080 |
| Diabetes [n (%)] | 14 (20.90%) | 5 (38.46%) | 9 (16.67%) | 0.175 |
| CA [n (%)] | 5 (7.46%) | 1 (7.69%) | 4 (7.41%) | 1.000 |
| SCD family history | 22 (32.84%) | 3 (23.08%) | 19 (35.18%) | 0.613 |
| History of syncope | 8 (11.94%) | 1 (7.69%) | 7 (12.96%) | 0.960 |
| SCD score | 4.89±2.26 | 4.53±1.99 | 4.98±2.33 | 0.525 |
| **6MWT (m)** | **369.40±59.64** | **338.46 ±65.04** | **376.85±56.40** | **0.036*** |
| HR1 (beats/min) | 73.63±9.09 | 73.77±9.09 | 73.59±9.18 | 0.950 |
| HR2 (beats/min) | 76.81±5.36 | 75.38±5.52 | 77.15±5.32 | 0.291 |
| SBP (mmHg) | 115.48±7.06 | 115.46±8.19 | 115.48±6.84 | 0.993 |
| DBP (mmHg) | 75.88±7.25 | 77.69±6.43 | 75.44±7.42 | 0.319 |
| β-blocker (metoprolol) |  |  |  |  |
| Non β-blocker | 6 (8.96%) | 1 (7.69%) | 5 (9.26%) | 1.000 |
| 47.5 mg qd | 3 (4.48%) | 1 (7.69%) | 2 (3.70%) | 1.000 |
| 90.0 mg qd | 3 (4.48%) | 0 (0.00%) | 3 (5.55%) | 0.902 |
| ≤6 (months) [n (%)] | 16 (23.88%) | 4 (30.75%) | 12 (22.22%) | 0.774 |
| 47.5 mg qd | 6 (8.96%) | 2 (15.38%) | 4 (7.41%) | 0.716 |
| 90.0 mg qd | 10 (14.92%) | 2 (15.38%) | 8 (14.82%) | 1.000 |
| 6~24 (months) [n (%)] | 23 (34.33%) | 5 (38.46%) | 18 (33.33%) | 0.981 |
| 47.5 mg qd | 14 (20.90%) | 3 (23.08%) | 11 (20.37%) | 1.000 |
| 90.0 mg qd | 9 (13.43%) | 2 (15.38%) | 7 (12.96%) | 1.000 |
| ≥24 (months) [n (%)] | 22 (32.84%) | 3 (23.08%) | 19 (35.18%) | 0.613 |
| 47.5 mg qd | 6 (8.96%) | 0 (0.00%) | 6 (11.11%) | 0.472 |
| 90.0 mg qd | 16 (23.88%) | 3 (23.08%) | 13 (24.07%) | 1.000 |
| WBC count (10^9^/L) | 5.39±1.16 | 5.57±1.34 | 5.34±1.12 | 0.524 |
| RBC count (10^12^/L) | 5.13±1.24 | 5.04±1.34 | 5.15±1.23 | 0.926 |
| HB (g/L) | 141.12±11.40 | 141.38±11.41 | 141.06±11.50 | 0.766 |
| PLT count (10^12^/L) | 205.69±52.87 | 210.46±58.67 | 204.54±51.92 | 0.720 |
| ALT (U/L) | 29.33±11.96 | 25.08±9.96 | 30.35±12.25 | 0.155 |
| AST (U/L) | 26.24±11.73 | 31.69±9.57 | 24.93±11.90 | 0.061 |
| TBiL (umol/L) | 10.42±5.37 | 9.81±6.25 | 10.57±5.19 | 0.652 |
| DBiL (umol/L) | 4.01±1.21 | 3.64±1.23 | 4.11±1.20 | 0.216 |
| K^+^ (mmol/L) | 4.24±0.45 | 4.25±0.52 | 4.24±0.44 | 0.949 |
| Na^+^ (mmol/L) | 141.06±3.97 | 142.54±4.65 | 140.70±3.75 | 0.136 |
| Cl^-^ (mmol/L) | 102.25±3.84 | 102.15±4.04 | 102.28±3.82 | 0.918 |
| Ca^2+^ (mmol/L) | 2.16±0.09 | 2.17±0.09 | 2.16±0.09 | 0.891 |
| GLU (mmol/L) | 6.01±0.58 | 6.24±0.49 | 5.96±0.59 | 0.113 |
| CREA (umol/L) | 78.37±10.15 | 76.66±9.73 | 78.78±10.29 | 0.502 |
| UA (umol/L) | 284.42±93.87 | 308.15±103.17 | 278.70±91.61 | 0.314 |
| UREA (mmol/L) | 5.98±1.80 | 5.14±1.74 | 6.18±1.77 | 0.060 |
| TG (mmol/L) | 1.12±0.31 | 1.16±0.35 | 1.11±0.30 | 0.661 |
| TC (mmol/L) | 3.64±0.73 | 3.71±0.67 | 3.62±0.74 | 0.701 |
| HDL (mmol/L) | 1.42±0.28 | 1.51±0.27 | 1.40±0.28 | 0.203 |
| LDL (mmol/L) | 2.54±0.28 | 2.49±0.22 | 2.55±0.29 | 0.465 |
| ApoA1 (g/L) | 1.29±0.24 | 1.31±0.22 | 1.28±0.24 | 0.663 |
| ApoB (g/L) | 1.07±0.19 | 1.06±0.19 | 1.07±0.19 | 0.846 |
| CK-MB (ng/ml) | 3.47±1.97 | 3.15±2.14 | 3.54±1.95 | 0.528 |
| TnI (ng/ml) | 0.03±0.03 | 0.02±0.02 | 0.03±0.03 | 0.625 |
| MB (ng/ml) | 60.68±25.59 | 51.46±21.92 | 62.90±26.09 | 0.149 |
| PT (sec) | 12.44±0.81 | 12.22±0.81 | 12.49±0.81 | 0.271 |
| PTR | 1.05±0.11 | 1.00±0.06 | 1.07±0.12 | 0.047 |
| PTINR | 1.21±0.37 | 1.13±0.13 | 1.22±0.40 | 0.413 |
| APTT (sec) | 34.75±4.71 | 34.15±5.05 | 34.89±4.66 | 0.617 |
| Fg (g/L) | 2.94±0.56 | 2.99±0.66 | 2.92±0.54 | 0.706 |
| TT (ses) | 17.72±2.05 | 16.90±2.11 | 17.91±2.00 | 0.109 |
| FDP (mg/L) | 3.25±1.18 | 3.03±1.09 | 3.30±1.20 | 0.456 |
| DD (mg/L) | 0.25±0.16 | 0.25±0.19 | 0.25±0.16 | 0.875 |
| TSH (uIU/ml) | 3.43±1.56 | 3.54±1.30 | 3.40±1.63 | 0.781 |
| FT3 (pmol/L) | 5.05±1.23 | 5.40±1.32 | 4.97±1.20 | 0.256 |
| FT4 (pmol/L) | 15.24±3.52 | 14.74±3.02 | 15.37±3.65 | 0.567 |
| BNP (pg/ml) | 144.61±60.39 | 161.31±49.95 | 140.59±62.38 | 0.270 |
| HbAlc (%) | 5.98±1.20 | 6.12±1.19 | 5.95±1.21 | 0.658 |
| **RVD1 (mm)** | **22.84±2.68** | **26.85±1.82** | **21.87±1.82** | **<0.001***** |
| **RVD2 (mm)** | **25.46±2.44** | **28.46±2.67** | **24.74±1.75** | **<0.001***** |
| LAD1 (mm) | 39.22±4.55 | 40.08±3.93 | 39.02±4.70 | 0.456 |
| LAD2 (mm) | 39.37±7.47 | 39.15±7.06 | 39.43±7.63 | 0.907 |
| SV (ml) | 50.46±6.23 | 49.31±6.34 | 50.74±6.23 | 0.461 |
| LVEF (%) | 59.24±4.51 | 60.54±4.27 | 58.93±4.55 | 0.250 |
| IVS (mm) | 26.13±5.25 | 24.46±6.29 | 26.54±4.94 | 0.203 |
| LVPW (mm) | 11.88±1.88 | 11.77±1.83 | 11.91±1.91 | 0.814 |
| LVM (g) | 326.13±75.59 | 311.69±79.01 | 329.61±75.09 | 0.447 |
| LVMI (g/m^2^) | 211.27±44.40 | 197.11±44.63 | 214.68±44.08 | 0.202 |
| Peak E (cm/s) | 83.16±13.27 | 86.00±10.73 | 82.48±13.81 | 0.395 |
| Peak A (cm/s) | 47.76±10.54 | 49.54±8.27 | 47.33±11.04 | 0.502 |
| E/A | 1.82±0.49 | 1.76±0.35 | 1.84±0.51 | 0.610 |
| IVS-e' (cm/s) | 5.54±0.86 | 5.61±0.75 | 5.52±0.89 | 0.727 |
| Lat-e' (cm/s) | 7.04±1.06 | 6.92±1.20 | 7.06±1.03 | 0.655 |
| E/e' | 13.67±3.83 | 12.62±4.33 | 13.93±3.70 | 0.271 |
| TRVmax (m/s) | 2.90±0.44 | 3.03±0.43 | 2.87±0.44 | 0.241 |
| **LAVI (ml/m^2^)** | **37.79±4.42** | **44.15±3.26** | **36.26±3.09** | **<0.001***** |
| LOVTG1 (mmHg) | 22.18±9.32 | 23.08±9.46 | 21.96±9.36 | 0.702 |
| LOVTG2 (mmHg) | 38.72±10.29 | 35.69±10.28 | 39.44±10.25 | 0.241 |
| LVOT obstruction |  |  |  |  |
| Non-obstruction | 13 (19.40%) | 4 (30.77%) | 9 (16.67%) | 0.445 |
| Occult-obstruction | 37 (55.22%) | 5 (38.46%) | 32 (59.26%) | 0.297 |
| Obstruction | 17 (25.37%) | 4 (30.77%) | 13 (24.07%) | 0.886 |
| Vmax1 (cm/s) | 249.18±80.75 | 235.77±71.63 | 252.41±83.09 | 0.509 |
| Vmax2 (cm/s) | 381.82±75.34 | 388.23±74.73 | 380.28±76.10 | 0.735 |
| **IVS-QSb (ms)** | **71.25±9.17** | **80.92±3.86** | **68.93±8.54** | **<0.001***** |
| **IVS-QSt (ms)** | **99.73±13.38** | **118.54±9.31** | **95.20±9.87** | **<0.001***** |
| **Lat-QSb (ms)** | **84.96±7.18** | **91.77±7.55** | **83.28±6.08** | **<0.001***** |
| **Lat-QSt (ms)** | **116.18±11.28** | **130.00±6.01** | **112.85±9.06** | **<0.001***** |
| P (ms) | 102.66±8.60 | 101.77±8.62 | 102.87±8.66 | 0.682 |
| QRS (ms) | 103.15±9.24 | 103.23±9.77 | 103.13±9.21 | 0.972 |
| PR (ms) | 148.81±18.38 | 151.46±18.71 | 148.17±18.42 | 0.566 |
| QT (ms) | 388.48±22.68 | 381.54±19.07 | 390.15±23.31 | 0.222 |
| QTc (ms) | 475.81±57.96 | 469.38±65.71 | 477.35±56.51 | 0.660 |
| QRS axis | 7.03±50.10 | 13.15±49.01 | 5.56±50.70 | 0.627 |
| RV_5_+SV_1_ (mv) | 2.94±0.60 | 2.88±0.59 | 2.95±0.61 | 0.693 |
| Total HR (beats) | 76444.19±3145.74 | 76564.46±2976.34 | 76415.24±3211.35 | 0.879 |
| Average HR (beats/min) | 69.36±6.71 | 68.85±6.07 | 69.48±6.90 | 0.762 |
| Minimum.HR (beats/min) | 61.13±10.27 | 59.69±8.95 | 61.48±10.61 | 0.577 |
| Maximum.HR (beats/min) | 100.96±6.28 | 101.08±4.87) | 100.93±6.62 | 0.939 |
| NSVT (frequency) | 24.76±14.56 | 22.85±12.32 | 25.22±15.12 | 0.601 |

Abreviations: HF: heart failure; NHF: none-heart failure; HF: heart failure; NHF: none-heart failure; BMI: body mass index; CA: coronary atherosclerosis (coronary artery stenosis <50%); SCD, sudden cardiac death; 6 MWT: 6 minutes walk test; HR1, heart rate at enrolment; HR2, heart rate during measurement; SBP, systolic blood pressure; DBP, diastolic blood pressure; WBC, white blood cell; RBC, red blood cell; Hb, haemoglobin; PLT, platelet; ALT, alanine aminotransferase; AST, aspartate aminotransferase; TBiL: total bilirubin; DBiL:direct bilirubin; K^+^: Kalium ion; Na^+^: sodium ion; Cl^-^: chlorine ion; Ca^2+^: calcium ion; GLU, glucose; CREA, creatinine; UA, uric acid; TG, triglyceride; TC, total cholesterol; HDL, high-density lipoprotein; LDL, low-density lipoprotein; ApoA1: apoli-poproteinA1; ApoB, apolipoprotein B; CK-MB, creatine kinase isoenzyme; TnI, troponin I; MB: myoglobin; PT: prothrombin time; PTR: prothrombin time ratio; PT-INR: prothrombin time-International normalized ratio; APTT, activated partial thromboplastin time; Fg, fibrinogen; TT, thrombin time; FDP, fibrinolytic products; DD, d-dimer; TSH, thyroid stimulating hormone; FT3, free T3; FT4, free T4; BNP, brain natriuretic peptide; HbAlc: glycated haemoglobin; RVD1, anteroposterior diameter of the right ventricle; RVD2, right ventricular transversal diameter; LAD1, anteroposter left atrial diameter; LAD2, left atrial dimension; SV, stroke volume; LVEF, left ventricular ejection fraction; LVPW: left ventricular posterior wall; LVM: left ventricular mass; LVMI: left ventricular mass index; E/A: Peak E/Peak A; IVS-e': interventricular septum-e'; Lat-e': lateral wall of left ventricle e '; E/e': mitral valve annulus tissue movement, E/e': TRVmax, tricuspid regurgitation peak velocity max; LAVI, left atrial volume index; LOVTG1, left ventricular outflow tract gradient at rest; LOVTG2: Left ventficular outflow tract gradient when performing the Valsalva manoeuvre; Vmax1: maximum velocity of the left ventficular outflow tract at rest; Vmax2: maximum velocity of the left venticular outflow tract When performing the Valsalva manoeuvre, IVS: intervicular septum, Lat: lateral wall, QSb: from the onset of QRS wave on ECG to the beginning of S wave on TDI; QSt: from the onset of the QRS wave on ECG to the top of the S wave on TDI. Total HR: 24-hour total heart rate; Average HR: 24-hour average heart rate; Minimum HR: Minimum heart rate in 24 hours HR: Maximum heart rate in 24 h; NSVT: non-sustained ventricular tachycardia; * P<0.05, ** P<0.01, ***P<0.001.


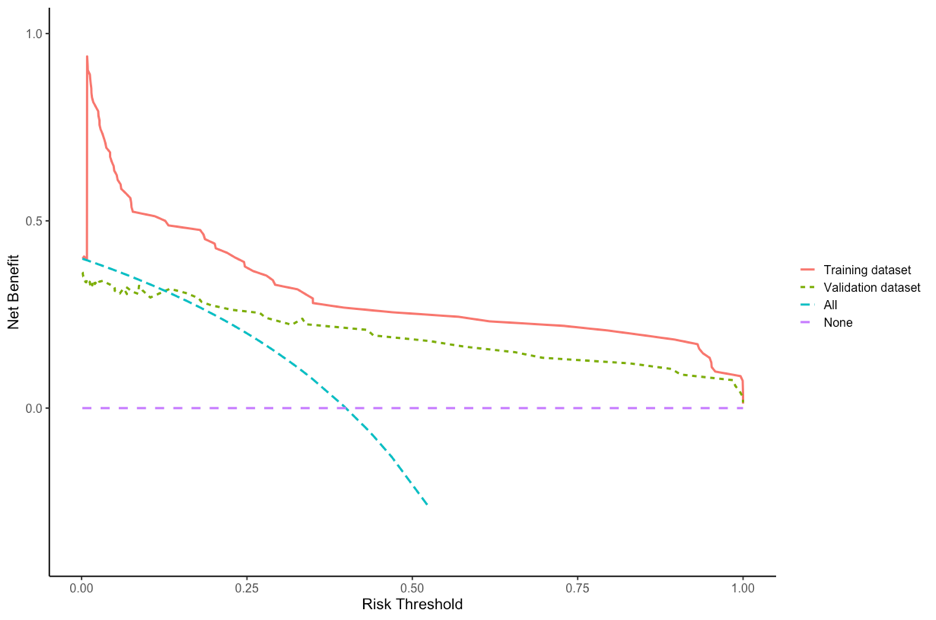
***Supplementary Figure 1***

***R codes:***

#1.Baseline data analysis

#1.1 step1 Download and load the R package

#1.1.1 install.packages

install.packages("tableone")

#1.1.2 load the package

library(tableone)

#1.1.3 Load Dataset Load your own dataset here

HCMorigin <- read.csv("D:\\Users\\wuyouzhenzheng\\Desktop\\HCMorigin.CSV", header=TRUE, na.strings=c("NA"))

***Supp Fig*.** Decision curve analysis of the predictive nomogram for predicting the probabilities of heart failure in training dataset and validation dataset respectively.

***R codes:***

#1.Baseline data analysis

#1.1 step1 Download and load the R package

#1.1.1 install.packages

install.packages("tableone")

#1.1.2 load the package

library(tableone)

#1.1.3 Load Dataset Load your own dataset here

HCMorigin <- read.csv("D:\\Users\\wuyouzhenzheng\\Desktop\\HCMorigin.CSV", header=TRUE, na.strings=c("NA"))

#1.1.4 View the variable names of the dataset

colnames(HCMorigin)

#1.1.5 Summarize a single group Summarize the entire dataset

CreateTableOne(data = HCMorigin)

#1.2 Specify the variables to be aggregated

myVars <- c("Age", "BMI", "Male", "Smoking", "Drinking","Hypertension",

"CA", "Diabetes", "HR.1", "SBP","DBP", "X6.minsTEST", "No.medication",

"X6.months", "X6.24months", "X24.months","WBC", "RBC", "HB", "PLT", "ALT", "AST","TBiL","DBiL", "K","Na","Cl","Ca", "GLU","CREA", "UA",

"UREA", "TG","TC","HDL","LDL","ApoAl","ApoB","CKMB","TnIDx", "MB", "PTT","PTR","PTINR","APTT","Fg","TT","FDP","DD","TSH","FT3", "FT4", "BNP", "HbAlc","RVD1","RVD2","LAD1","LAD2","SV","LVEF", "IVS",

"LVPW", "LVM","LVMI", "HR2","Peak.E","Peak.A", "E.A","IVS.e","Lat.e","E.e","TRVmax","LAVI","LOVTG1", "LOVTG2","Vmax1","Vmax2","P","QRS","PR","QT","QTc", "electric.axis","RV5andSV1","Total.heart.beats","Average.heart.rate","The.slowest.heart.rate","The.fastest.heart.rate","IVSQSb","IVSQSt","LatQSb","LatQSt", "NSVT")

#1.3 specify which are categorical variables

catVars <- c( "Male","Smoking", "Drinking", "Hypertension", "CA", "Diabetes", "No.medication","X6.months","X6.24months","X24.months")

#1.4 Optimizing Single Group Summary Tables

tab2 <- CreateTableOne(vars = myVars, data =HCMorigin, factorVars = catVars)

tab2

#1.5 To display data for all levels, enter:

print(tab2, showAllLevels = TRUE)

#1.6 Show all data information

summary(tab2)

#1.7 Whether there is a heart failure event (1 for HF, 0 for no HF) needs to be grouped and summarized.

tab3 <- CreateTableOne(vars = myVars, strata = "HF" , data =HCMorigin, factorVars = catVars)

tab3

#2.Unit COX regression analysis with or without concomitant heart failure as an event

#2.1 Load these three R packages

install.packages("forestplot")

library("survival")

library("survminer")

#2.2 set random number seed

set.seed(4234)

set.seed(2351)

#2.3 Load the HCMorigin dataset

HCMorigin <- read.csv("D:\\Users\\wuyouzhenzheng\\Desktop\\HCMorigin.CSV", header=TRUE, na.strings=c("NA"))

dput(names(HCMorigin))

#Note that the coxph function in the survival package is used here

#Pay attention to adjusting the event name and time variable name

res.cox <- coxph(Surv(FUT,HF) ~ BNP , data = HCMorigin)

res.cox

summary(res.cox)

#The following is a univariate cox regression analysis for all characteristics

covariates <- c( "Age", "BMI", "Male", "Smoking", "Drinking", "Hypertension", "CA", "Diabetes", "HR.1", "SBP", "DBP", "X6MWT", "No.medication",

"X6.months", "X6.24months", "X24.months", "WBC", "RBC", "HB",

"PLT", "ALT", "AST", "TBiL", "DBiL", "K", "Na", "Cl", "Ca", "GLU",

"CREA", "UA", "UREA", "TG", "TC", "HDL", "LDL", "ApoAl", "ApoB",

"CKMB", "TnIDx", "MB", "PTT", "PTR", "PTINR", "APTT", "Fg", "TT",

"FDP", "DD", "TSH", "FT3", "FT4", "BNP", "HbAlc", "RVD1", "RVD2",

"LAD1", "LAD2", "SV", "LVEF", "IVS", "LVPW", "LVM", "LVMI", "HR2",

"Peak.E", "Peak.A", "E.A", "IVS.e", "Lat.e", "E.e","TRVmax",

"LAVI", "LOVTG1", "LOVTG2", "Vmax1", "Vmax2", "P", "QRS", "PR",

"QT", "QTc", "Electric.axis", "RV5andSV1", "Total.heart.beats",

"Average.heart.rate", "The.slowest.heart.rate", "The.fastest.heart.rate",

"IVSQSb", "IVSQSt", "LatQSb", "LatQSt","SCD.family.history",

"History.of.syncope", "SCDtest", "NSVT")

#2.4 For each variable separately, construct the formula for survival analysisuniv_formulas <- sapply(covariates,function(x) as.formula(paste('Surv(FUT,HF) ~', x)))

#2.5 Loop through cox regression analysis for each featureuniv_models <- lapply(univ_formulas, function(x){coxph(x, data =HCMorigin)})

#2.6 Extract HR, 95% confidence intervals and p-values

univ_results <- lapply(univ_models,

function(x){

x <- summary(x)

#2.6.1 get p-value

p.value<-signif(x$wald["pvalue"], digits=3)

#2.6.2 Get HR value

HR <-signif(x$coef[2], digits=3);

#2.6.3 Get the 95% confidence interval

HR.confint.lower <- signif(x$conf.int[,"lower .95"], 3)

HR.confint.upper <- signif(x$conf.int[,"upper .95"],3)

HR <- paste0(HR, " (", HR.confint.lower, "-", HR.confint.upper, ")")

res<-c(p.value,HR)

names(res)<-c("p.value","HR (95% CI for HR)")

return(res)

})

#2.6.4 Convert to data frame and transpose

res <- t(as.data.frame(univ_results, check.names = FALSE))

as.data.frame(res)

#3.Multivariate cox regression analysis

res.cox <- coxph(Surv(FUT,HF) ~ IVSQSb + IVSQSt + LatQSb + LatQSt + X6MWT + CREA + ApoAl + MB + RVD1 + RVD2 + LVEF + LAVI + P + QRS + PR + SCD.family.history, data = HCMorigin)

x <- summary(res.cox)

pvalue=signif(as.matrix(x$coefficients)[,5],2)

HR=signif(as.matrix(x$coefficients)[,2],2)

low=signif(x$conf.int[,3],3)

high=signif(x$conf.int[,4],4)

multi_res=data.frame(p.value=pvalue,

HR=paste(HR," (",low,"-",high,")",sep=""),

stringsAsFactors = F)

multi_res

Summary(res.cox)

#4.Calculate C-index

library(Hmisc)

cindex =1-rcorr.cens(predict(fit),Surv(as.numeric(HCMorigin$FUT),HCMorigin$HF))

C-index

#5.Ploting the nomogram.

#Loading the required R packages "survival, rms"

library("survival")

library("rms")

#Loading database HCMorigin

HCMorigin <- read.csv("D:\\Users\\wuyouzhenzheng\\Desktop\\HCMorigin.CSV", header=TRUE, na.strings=c("NA"))

#setting random number seed

set.seed(1234)

set.seed(2351)

#conversion database

datas=read.csv("D:\\Users\\wuyouzhenzheng\\Desktop\\HCMorigin.CSV", header=T, na.strings=c("NA"))

dd=datadist(datas)

option <- options(datadist="dd")

#Building a multivariate COX regression model

MultiCOX5 <- cph(Surv(FUT,HF) ~ LatQSb + LatQSt + MB + LAVI + PR, data = datas,x=TRUE,y=TRUE,surv = TRUE, time.inc=60)

surv1 = function(x)surv(18, x)

surv2 = function(x)surv(24, x)

med <- Quantile(MultiCOX5)

nom <- nomolung = nomogram(MultiCOX5,

fun = list(

surv1, surv2), lp=F,

fun.at = c(0.05, seq(0.1, 0.9, by = 0.4), 0.95),

funlabel = c('13 month survial', '34 month survival'))

nom <- nomogram(MultiCOX5, fun=function(x) med(lp=x),

funlabel="Median Survival Time")

#Output nomogram graphics

plot(nom)

#6.Calculate the score and cutoff value of the nomogram for each sample

#Use the formula_lp() function and the points_cal() function in the nomogramFormula package

#Directly extract the linear predictors in the data set linear.predictors, calculate the nomogram score

options(option)

results <- formula_lp(nomogram = nom)

points <- points_cal(formula = results$formula, lp = MultiCOX5$linear.predictors)

summary(points)

#Get the nomogram's score for each sample.

#Calculate the cutoff value of nomogram's score

#Load the following packages

library("ggpubr")

library("broom")

library("ggplot2")

library("magrittr")

library("survival")

library("survminer")

library("graphics")

#load database

nomogramscore <- read.csv("D:\\Users\\wuyouzhenzheng\\Desktop\\nomogramscore.CSV", header=T, na.strings=c("NA"))

# View dataset

View(nomogramscore)

HF <- nomogramscore[,1]

FUT <- nomogramscore[, 2]

score1 <- nomogramscore[,3]

set.seed(1234)

set.seed(2351)

colnames(nomogramscore)

clinical_info <- nomogramscore

colnames(clinical_info)

res.cut <- surv_cutpoint(clinical_info, time = "FUT", event ="HF" ,

variables = c("score1"))

summary(res.cut)

a<-summary(res.cut)

a

res.cat <- surv_categorize(res.cut)

head(res.cat)

aidatamerge <- merge.data.frame(KM,res.cat, by = "HF", all = T,sort = T)

colnames(aidatamerge)

write.csv(aidatamerge, file = "D:\\Users\\wuyouzhenzheng\\Desktop\\KM.csv")

#7.According to the cutoff value of the nomogram score, the upper and lower groups are grouped, and the km curve is drawn.

#Build the dataset KM

#Draw a KM map. Note that the km map can only be used for categorical variables, not continuous variables.

library("survival")

library("survminer")

rm(list=ls())

KM <- read.csv("D:\\Users\\wuyouzhenzheng\\Desktop\\KM.CSV", header=T, na.strings=c("NA"))

View(KM)

HF <- KM[,1]

FUT <- KM[, 2]

score <- KM[,3]

cutoff <- KM[,4]

Surv(FUT,HF) # Create a survival object

attach(KM) # bind dataset

fit <- survfit(Surv(FUT,HF) ~ cutoff, data = KM)

fit # View Fit Curve Information

summary(fit)

ggsurvplot(fit, data = KM)

ggsurvplot(fit, data = KM,

surv.median.line = "hv") # Increase median survival time

ggsurvplot(fit, data = KM,

#conf.int = TRUE, # Increase the confidence interval

fun = "cumhaz") # Plot the cumulative risk curve

ggsurvplot(fit, data = KM,

conf.int = TRUE, # Increase the confidence interval

risk.table = TRUE) # Plot the cumulative risk curve

ggsurvplot(fit, # Created fit object

data = KM, # Specify variable data source

conf.int = TRUE, # Show confidence intervals

pval = TRUE, # Add P value

surv.median.line = "hv", # Add median survival timeline

add.all = TRUE) # Add Overall Patient Survival Curve

#custom color palette

ggsurvplot(fit, # Created fit object

data = KM, # Specify variable data source

conf.int = TRUE, # Show confidence intervals

pval = TRUE, # Add P value

surv.median.line = "hv", # Add median survival timeline

palette = "hue") # Add Overall Patient Survival Curve

#Optional palettes are "grey","npg","aaas","lancet","jco",

#"ucscgb","uchicago","simpsons"和"rickandmorty".

#Beautify the Survival Curve

ggsurvplot(fit, # Created fit object

data = KM, # Specify variable data source

conf.int = TRUE, # Show confidence intervals

pval = TRUE, # Add P value

pval.coord=c(0.45,0.30),

#surv.median.line = "hv", # Add median survival timeline

risk.table = TRUE, # Add risk table

xlab = "Follow up time(months)", # specify x-axis labels

ylab = "Free of heart failure probability",

legend = c(0.12,0.12), # Specify the legend position

legend.title = " ", # Set legend title

legend.labs = c("score<230.65","score≥230.65"), # Specify legend grouping labels

break.y.by = 0.1,

break.x.by = 2,# Set the x-axis tick spacing

palette = "lancet")

#7.1 log-rank test

surv_diff <- surv_diff(Surv(FUT,HF) ~ cutoff, data = KM)

print(surv_diff)

#8.DCA cox regression in primary dataset and validation dataset

library(ggDCA)

library(caret)

library(rmda)

library(rms)

setwd("~/dataset/zhaona")

data1 <-read.csv("DCA.csv")

clinical_info <- data1

clinical_info<-datadist(clinical_info)

options(datadist= clinical_info )

clinical_infotrain<-datadist(clinical_infotrain)

options(datadist= clinical_infotrain)

set.seed(123)

source("stdca.R")

clinical_info$Time_death <- clinical_info$FUT

clinical_info$Status_death <- clinical_info$HF

clinical_infotrain$Time_death <- clinical_infotrain$FUT

clinical_infotrain$Status_death<-clinical_infotrain$HF

Srv <- Surv(clinical_info$Time_death, clinical_info$Status_death)

Srv2 <- Surv(clinical_infotrain$Time_death, clinical_infotrain$Status_death)

coxmod1 = coxph(Srv ~ LatQSt + LatQSb + MB + LAVI + PR, data=clinical_info)

coxmod2 = coxph(Srv2 ~ LatQSt + LatQSb + MB + LAVI + PR, data=clinical_infotrain)

#the probability of failure is calculated by subtracting the probability of

#survival from 1.

clinical_info$pr_failure18 <- c(1- (summary(survfit(coxmod1,

newdata=clinical_info), times=34)$surv))

clinical_infotrain$pr_failure19 <- c(1- (summary(survfit(coxmod2,

newdata=clinical_infotrain), times=34)$surv))

#Run the decision curve analysis (with a smoother)

stdca(data=clinical_info, outcome="Status_death", ttoutcome="Time_death", timepoint=34,

predictors="pr_failure18",

xstop=0.5, smooth=TRUE)

stdca(data=clinical_infotrain, outcome="Status_death", ttoutcome="Time_death", timepoint=34,

predictors="pr_failure19",

xstop=0.5, smooth=TRUE)

median(clinical_info$Time_death)

validate(m1, method="boot", B=1000, dxy=T) #重复模拟1000次

rcorrcens(Surv(FUT,HF) ~ predict(m2), data = clinical_info)

#9.calibration curve of External validation dataset

m18 <- cph(Surv(Time_death,Status_death)~LatQSb + LatQSt + MB + LAVI + PR,

data = clinical_info, time.inc = 34, x=T, y=T, surv=T)

m19 <- cph(Surv(Time_death,Status_death)~LatQSb + LatQSt + MB + LAVI + PR,

data = clinical_infotrain, time.inc = 34, x=T, y=T, surv=T)

cal3 <- calibrate(m19, cmethod="KM", method="boot", u=26, m=19, B= 200)

plot(cal3,

xlab = "Predicted with non-HF",

ylab = "Fraction with non-HF")

#10.ICC

library(irr)

clinical_info <- data2

colnames(clinical_info)

vars <- colnames(clinical_info)[c(90:93)]

corr1 <- clinical_info %>%

dplyr::select(vars)

icc(corr1, model="twoway", type = "consistency",

unit = "average")
